# Surgical outcomes of pediatric brain tumors in Sub-Saharan Africa: A systematic review

**DOI:** 10.1016/j.bas.2022.100912

**Published:** 2022-07-03

**Authors:** Vendela Herdell, Philipp Lassarén, Frederick A. Boop, Jiri Bartek, Enoch O. Uche, Magnus Tisell

**Affiliations:** a Karolinska Institutet, Stockholm, Sweden; b Department of Global Pediatric Medicine, St. Jude Children's Research Hospital, Memphis, TN, USA; c Department of Neurosurgery, University of Tennessee Health Science Center, Memphis, TN, USA; d Department of Neurosurgery, Karolinska University Hospital, Stockholm, Sweden; e Department of Clinical Neuroscience, Section of Neurosurgery, Karolinska Institutet, Stockholm, Sweden; f Department of Neurosurgery, Rigshospitalet, Copenhagen, Denmark; g Division of Neurosurgery, Department of Surgery, University of Nigeria Nsukka, Ituku-Ozalla Campus, Enugu, Nigeria; h Department of Surgery, University of Nigeria Teaching Hospital, Ituku/Ozalla, Enugu, Nigeria; i Department of Neurosurgery, Sahlgrenska University Hospital, Gothenburg, Sweden; j Department of Neuroscience, Sahlgrenska Academy, University of Gothenburg, Gothenburg, Sweden

**Keywords:** Sub-Saharan Africa, Pediatric, Brain tumor, Neurosurgery, Outcomes

## Abstract

**Background:**

Pediatric Brain Tumors (PBT) are a common cause of cancer-related mortality globally. Contrary to high-income countries (HIC), survival rates in low-and-middle income countries (LMIC) remains low despite advances in neurosurgical care and diagnostics over the past decades. The aim of this systematic review was to investigate the surgical outcomes for PBT in Sub-Saharan Africa, and the distribution of PBT types.

**Methods:**

A systematic review was conducted on PubMed, for all available literature on the surgical outcomes of PBT in Sub-Saharan Africa, published before May 3, 2022. Two reviewers performed abstract, full text screening and data collection independently, resolving any conflicts by consensus.

**Results:**

The search yielded 256 studies, of which 22 met the inclusion criteria, amounting to a total of 243 patients. Nigeria was the country with most data. Only subgroups of patients could be extracted from 12 studies, and variables of interest in 6 studies had inconsistent sample sizes. The age centered around 9 years, and there were approximately equal number of girls and boys. The most common tumor was medulloblastoma, followed by craniopharyngioma and astrocytoma. There was large heterogeneity in the reporting of outcomes, and a trend was difficult to discern, considering the large number of different tumor types and different extents of resection.

**Discussion and conclusion:**

Data is insufficient and inconsistent, precluding statistical conclusions. There is a need for more studies in the field.

## Highlights

• Data on outcomes of pediatric brain tumor surgery in Sub-Saharan Africa is insufficient, preventing statistical conclusions.
• Nigeria is over-represented among studies on pediatric brain tumor and associated surgical outcomes in Sub-Saharan Africa.
• Medulloblastoma was the most commonly reported pediatric brain tumor in surgical outcome studies in Sub-Saharan Africa.

## Abbreviations:

HIC: high-income country
LMIC: low- and middle-income country
PBT: pediatric brain tumor

## 1 Background

Pediatric brain tumors (PBT) are the most common cause of pediatric cancer related mortality worldwide (Adel Fahmideh and Scheurer, 2021; Pollack, 1994). Yet, advances in diagnostics and treatment in high-income countries (HIC) over the past decades have resulted in relatively high survival rates (Allemani et al., 2018). Unfortunately, this is not the case in low and middle-income countries (LMIC) (Allemani et al., 2018), where 90% of the pediatric population lives (Bhakta et al., 2019). Often, prevalence and incidence of PBT in these countries appear low (Stoeter et al., 2021), which has been suggested to be due to underdiagnosis (Stagno et al., 2014), and lack of surgical treatment (Dewan et al., 2018). The first line treatment for most PBT is neurosurgical resection of the tumor (Pollack, 1994), and the extent of the brain tumor resection may depend on the tumor type (Grewal et al., 2020). However, data show a deficit in neurosurgeons compared to neurosurgical burden, especially in Sub-Saharan Africa (Dewan et al., 2018). The research material on PBT in Sub-Saharan Africa is scarce (Uche et al., 2021), but does provide important information on the current situation. One systematic review has been performed on abandonment of treatment for children with PBT in LMIC (Seah et al., 2019), and several attempts at mapping PBT in LMIC have been done (Allemani et al., 2018; Bhakta et al., 2019). Yet, a systematic review on the existing studies on PBT surgical outcomes in the Sub-Saharan African region is lacking.

The overall aim of this systematic review was to investigate the outcomes following surgery for PBT in Sub-Saharan Africa. More specifically, the following questions were posed:

a. What are the surgical outcomes for PBT in the Sub-Saharan African region, such as survival rates, postoperative recovery, and postoperative complications?
b. What types of PBT are included in the studies on surgical outcomes for PBT in the Sub-Saharan African region?

## 2 Methods

### 2.1 Search strategy and data selection

A systematic review was conducted in accordance with the Preferred Reporting Items for Systematic Reviews and Meta-Analyses (PRISMA) statement (Page et al., 2021). The search was performed on PubMed for all available literature on the surgical outcomes of PBT in Sub-Saharan Africa published before May 3, 2022 (Table 1). Two authors, VH and PL, performed abstract and full text screening independently, resolving any conflicts by consensus.

**Table 1:** PubMed search string.

(Angola OR Benin OR Botswana OR Burkina Faso OR Burundi OR Cabo Verde OR Cameroon OR Central African Republic OR Chad OR Comoros OR Congo OR Congo, Dem. Rep. OR Congo, Rep. OR Côte d'Ivoire OR Equatorial Guinea OR Eritrea OR Eswatini OR Ethiopia OR Gabon OR Gambia, The OR Ghana OR Guinea OR Guinea-Bissau OR Ivory coast OR Kenya OR Lesotho OR Liberia OR Madagascar OR Malawi OR Mali OR Mauritania OR Mauritius OR Mozambique OR Namibia OR Niger OR Nigeria OR Rwanda OR São Tomé and Principe OR Senegal OR Seychelles OR Sierra Leone OR Somalia OR South Africa OR South Sudan OR Sudan OR Tanzania OR Togo OR Uganda OR Zambia OR Zimbabwe)
*AND*
*(Glioma OR Astrocytoma OR Ganglioglioma OR Subependymoma OR Ependymoma OR Oligodendroglioma OR Glioblastoma OR Gliosarcoma OR Gliomatosis cerebri OR Meningioma OR Medulloblastoma OR Ependymoblastoma OR Pineoblastoma OR Pituitary adenoma OR Pituitary carcinoma OR Craniopharyngioma OR Rathke's cleft cyst OR Pineal Tumors OR Pineal cyst OR Pineocytoma OR Germinoma OR Pineal teratoma OR Choroid plexus tumors OR Choroid plexus papilloma OR Choroid plexus carcinoma OR Neurocytoma OR Dysembroplastic neuroepithelial tumor OR Hemangioblastoma OR Colloid cyst OR Arachnoid cysts OR Primary Central Nervous System Lymphoma OR Brain tumor OR Brain malignancy OR Cerebral tumor OR Cerebral malignancy)*
*AND*
*(Outcome OR Death OR Died OR Survival OR Survived OR Month OR Year)*
*AND*
*(Neurosurgery OR Surgical OR Surgery OR Operation OR Operative)*
*AND*
*(Pediatric OR Pediatrics OR Child OR Children OR Infants OR Adolescent OR Teenager)*

### 2.2 Eligibility criteria and study selection

Studies were included if they report outcomes after surgical intervention on patients aged 0–18 years with a histologically specified primary neoplastic intracranial brain tumor according to the 2021 5th edition of the World Health Organization (WHO) Classification of Tumors of the Central Nervous System (Central Nervous System Tumours, 2021). There was no restriction on the study design or language of publication. Studies with unavailable full text were excluded and listed in Supplementary File 1.

### 2.3 Data extraction and risk of bias assessment

Study and patient characteristics, including name of first author, year and country of publication, study type, number and age of patients, and tumor types were collected. Surgical outcomes, including surgical extent, survival/mortality data, length of hospital stay, quality-of-life, postoperative improvement and postoperative complications were collected. All data acquisition was performed independently by both screeners and cleared of conflicts. Data was collected on sample size for each variable of interest. Inconsistencies in the patient number between variables was used as a proxy for the relevant criteria in the Joanna Briggs Institute (JBI) Critical Appraisal Checklists for Case Reports and Case Series (Aromataris and Munn, 2020). Several inconsistences indicated more risk of bias. Furthermore, studies with large differences between number of patients of entire studies and relevant patients in this review also indicate a risk of bias, as well as the relevant sample size per se.

### 2.4 Data analysis

All data were first presented descriptively. Whenever possible, meta-analysis was performed in R (version 4.1.0) (Core Team, 2020) using the meta package (Balduzzi et al., 2019). Data were pooled with a random-effects model using the DerSimonian-Laird estimator for between-study variance (DerSimonian and Laird, 1986). Proportions were visualized using a forest plot. The *I*^2^ was used to quantify the between-study heterogeneity. Publication bias was assessed using Egger's (Egger et al., 1997) and Begg's (Begg and Mazumdar, 1994) tests, and funnel plots were drawn. No meta-regression was performed.

## 3 Results

### 3.1 Study characteristics

**Fig. 1:**
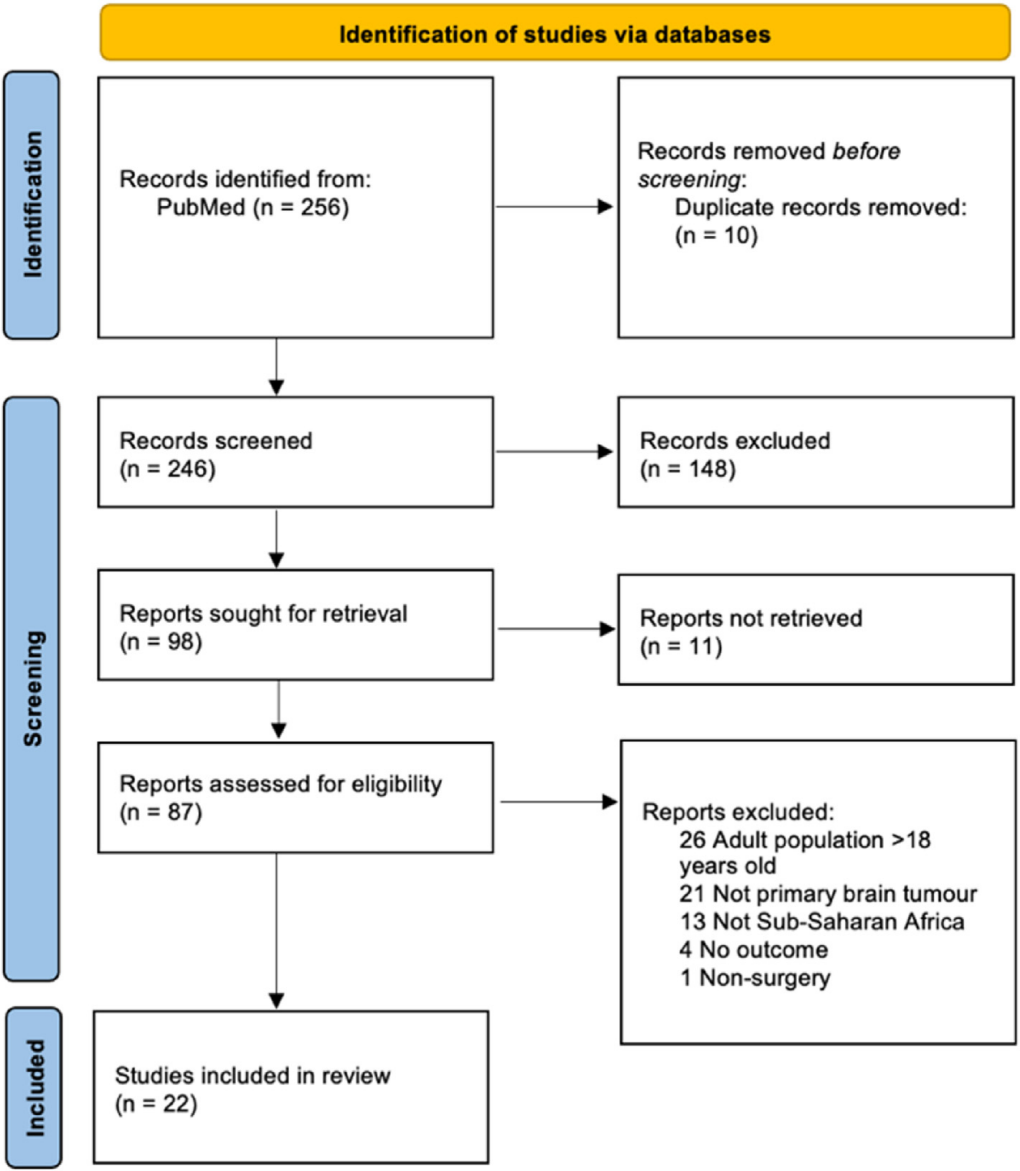
PRISMA flowchart.

**Fig. 2:**
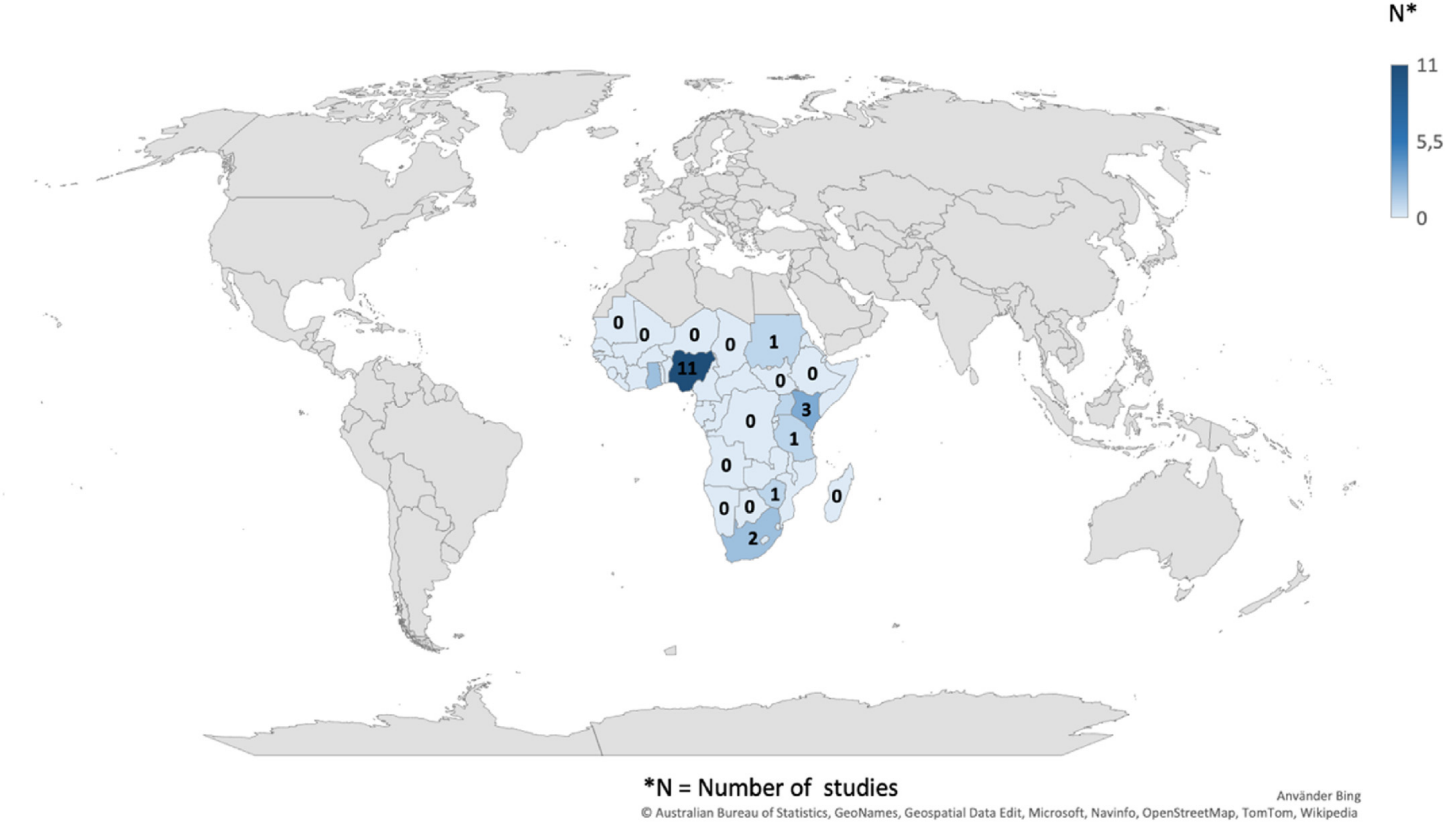
Map showing the distribution of studies in Sub-Saharan African countries.

There were 256 studies identified in the search, of which 22 studies (Uche et al., 2021; Adeloye et al., 1988; Adeolu et al., 2015; Andrews et al., 2003; Anunobi et al., 2016; Charles et al., 2019; Elhassan et al., 2019; Iddrissu et al., 2005; Kakusa et al., 2019; Labuschagne, 2020; Malomo et al., 2018; Mwang'ombe et al., 2002; Nadvi and van Dellen, 1994; Ndubuisi et al., 2018; Okechi and Albright, 2012; Olufemi Adeleye and Balogun, 2009; Onyia and Ojo, 2020; Salami et al., 2019; Seligson and Levy, 1974; Uche et al., 2013; Wanyoike, 2004; Wilson et al., 2012) met the inclusion criteria (Table 2). The included studies were case reports (n ​= ​9), retrospective chart reviews of cohorts (n ​= ​9), prospective observational cohort studies (n ​= ​2), a prospective treatment trial (n ​= ​1) and a combined retrospective chart review and prospective survey (n ​= ​1). Nigeria was the country with most abundant data (Fig. 2). Eleven studies were published in Nigeria, amounting in 185 patients. Three studies came from Kenya (32 patients), two from South Africa (12 patients) and Ghana (3 patients), and one from each of Uganda, Tanzania, Sudan, and Rhodesia (Fig. 1). Importantly, 12 studies contained only a subgroup of patients that met the inclusion criteria, effectively resulting in significantly fewer relevant patients than in the original studies (243 vs. 701 patients). Furthermore, there were 6 studies that had inconsistent number of patients for the relevant variables. The fraction of relevant patients, inconsistent number of patients and total patient number are indicators for the risk of bias.

**Table 2:** Study characteristics.

Study	Countries	Study design	Total study sample size	*De facto* sample size of subset	Sample size inconsistency in subset	The subset: relevant variable-specific sample size				
						Age	Sex	Tumor type	Extent of resection	Survival
Adeloye et al. (1988) (Adeloye et al., 1988)	Nigeria	Chart review	20	19	No	19	19	19	19	19
Adeolu et al. (2015) (Adeolu et al., 2015)	Nigeria	Case report	2	1	No	1	1	1	1	1
Andrews et al. (2003) (Andrews et al., 2003)	Ghana	Chart review	30	2	No	2	NA	2	2	2
Anunobi et al. (2016) (Anunobi et al., 2016)	Nigeria	Case report	1	1	No	1	1	1	1	1
Charles et al. (2019) (Charles et al., 2019)	Nigeria	Chart review	30	1	No	1	1	1	1	1
Elhassan et al. (2019) (Elhassan et al., 2019)	Sudan	Chart review	62	7	No	7	7	7	7	7
Iddrissu et al. (2005) (Iddrissu et al., 2005)	Ghana	Case report	1	1	No	1	1	1	1	1
Kakusa et al. (2019) (Kakusa et al., 2019)	Uganda	Chart review ​+ ​prospective survey	112	2	Yes	3	2	2	2	2
Labuschagne (2020) (Labuschagne, 2020)	South Africa	Prospective treatment trial	11	11	No	11	11	11	11	11
Malomo et al. (2018) (Malomo et al., 2018)	Nigeria	Case report	1	1	No	1	1	1	1	1
Mwang'ombe et al. (2002) (Mwang'ombe et al., 2002)	Kenya	Case report	1	1	No	1	1	1	1	1
Nadvi (1994) (Nadvi and van Dellen, 1994)	South Africa	Case report	1	1	No	1	1	1	1	1
Ndubuisi (2018) (Ndubuisi et al., 2018)	Nigeria	Chart review	54	45	Yes	54	45	46	45	45
Okechi (2012) (Okechi and Albright, 2012)	Kenya	Case report	1	1	No	1	1	1	1	1
Olufemi Adeleye (2009) (Olufemi Adeleye and Balogun, 2009)	Nigeria	Case report	1	1	No	1	1	1	1	1
Onyia (2020) (Onyia and Ojo, 2020)	Nigeria	Case report	1	1	No	1	1	1	1	1
Salami et al. (2019) (Salami et al., 2019)	Nigeria	Chart review	9	9	No	9	9	9	9	9
Seligson (1974) (Seligson and Levy, 1974)	Rhodesia	Chart review	153	1	Yes	18	NA	1	1	1
Uche et al. (2021) (Uche et al., 2021)	Nigeria	Prospective observational	92	76	Yes	92	92	78	76	76
Uche et al. (2013) (Uche et al., 2013)	Nigeria	Chart review	40	30	Yes	40	40	40	40	30
Wanyoike (2004) (Wanyoike, 2004)	Kenya	Chart review	37	30	Yes	37	37	29	33	30
Wilson et al. (2012) (Wilson et al., 2012)	Tanzania	Prospective observational	41	1	No	1	1	1	1	1

### 3.2 Patient characteristics

Among the 22 studies, the average reported ages ranged from 1 month to 16 years (Table 3), and the median thereof was 9 years. Among case reports, boys were more common (6 boys vs 3 girls), while girls were slightly more common in studies with more than three relevant patients (unweighted average 53%, weighted average 51%). The five most common tumor types were medulloblastoma (11 studies, 61 patients), craniopharyngioma (5 studies, 57 patients), astrocytoma (7 studies, 46 patients), meningioma (9 studies, 24 patients), and ependymoma (5 studies, 14 patients). The two most common reported extents of resection were [gross] total (8 studies, 93 patients), and subtotal (7 studies, 90 patients).

**Table 3:** Patient characteristics.

Study	Age, mean (range)	Sex, n female (%)	Tumor type	Surgical extent	Survival/mortality
Adeloye et al. (1988) (Adeloye et al., 1988)	9 ​y (Allemani et al., 2018; Bhakta et al., 2019; Stoeter et al., 2021; Stagno et al., 2014; Dewan et al., 2018; Grewal et al., 2020; Uche et al., 2021; Seah et al., 2019; Page et al., 2021; Central Nervous System Tumours, 2021; Aromataris and Munn, 2020; Core Team, 2020; Balduzzi et al., 2019)	8 (Foster et al., 2021)	Craniopharyngioma	Majority non-total resection	6 (32%) died (after 12 ​d - 6 ​m)
Adeolu et al. (2015) (Adeolu et al., 2015)	5 ​y	0 (0)	Craniopharyngioma	Near-total resection	NA
Andrews et al. (2003) (Andrews et al., 2003)	11 ​y	NA	Medulloblastoma	NA	1 (50%) died (after 3 ​d)
Anunobi et al. (2016) (Anunobi et al., 2016)	8 ​m	0 (0)	Clear cell meningioma	Subtotal resection	Patient died (after 6 ​h)
Charles et al. (2019) (Charles et al., 2019)	12 ​y	0 (0)	Medulloblastoma	Gross total resection	Patient died (after re-operation at 9 ​m)
Elhassan et al. (2019) (Elhassan et al., 2019)	10 ​y (Stoeter et al., 2021; Stagno et al., 2014; Dewan et al., 2018; Grewal et al., 2020; Uche et al., 2021; Seah et al., 2019; Page et al., 2021; Central Nervous System Tumours, 2021; Aromataris and Munn, 2020; Core Team, 2020)	3 (Park et al., 2021)	Astrocytoma (grade 1, n ​= ​2; grade 2, n ​= ​1) Meningioma (grade 1, n ​= ​1) Adenoma (n ​= ​1) Medulloblastoma (n ​= ​2)	Gross total resection (n ​= ​5) Subtotal resection (n ​= ​2)	Median survival 76 ​m (65–144)^b^
Iddrissu et al. (2005) (Iddrissu et al., 2005)	4 ​y	0 (0)	Anaplastic ependymoma	Subtotal resection ​+ ​shunt	Patient survived (at least 13 ​m)
Kakusa et al. (2019) (Kakusa et al., 2019)	11 ​y^a^ (Stoeter et al., 2021; Stagno et al., 2014; Dewan et al., 2018; Grewal et al., 2020; Uche et al., 2021; Seah et al., 2019; Page et al., 2021; Central Nervous System Tumours, 2021)	0 (0)	Medulloblastoma	Shunt	Median survival 6.9 ​m (0.03–13.8)
Labuschagne (2020) (Labuschagne, 2020)	4 ​y^a^ (Pollack, 1994; Allemani et al., 2018; Bhakta et al., 2019; Stoeter et al., 2021; Stagno et al., 2014; Dewan et al., 2018; Grewal et al., 2020; Uche et al., 2021; Seah et al., 2019; Page et al., 2021; Central Nervous System Tumours, 2021)	7 (64)	Astrocytoma grade IV (n ​= ​5) Anaplastic ependymoma (n ​= ​3) CNS embryonal tumor (n ​= ​2) Atypical teratoid/rhabdoid tumor (n ​= ​1)	Gross total resection (n ​= ​9) Non-total resection (n ​= ​2)	NA
Malomo et al. (2018) (Malomo et al., 2018)	12 w	0 (0)	Atypical choroid plexus papilloma	Gross total resection	NA
Mwang'ombe et al. (2002) (Mwang'ombe et al., 2002)	1 ​m	1 (100)	Fronto-ethmoidal teratoma	Total resection	NA
Nadvi (1994) (Nadvi and van Dellen, 1994)	16 ​y	0 (0)	Medulloblastoma	Near-total resection	NA
Ndubuisi (2018) (Ndubuisi et al., 2018)	9 ​y (0–17)	24 (53)	Glioma (n ​= ​20) Medulloblastoma (n ​= ​13) Craniopharyngioma (n ​= ​11) Meningioma (n ​= ​2)	Gross total resection (n ​= ​25) Subtotal resection (n ​= ​20)	18 (40%) died (within 1 ​y), distributed as 7/25 gross total resection and 11/20 subtotal resection
Okechi (2012) (Okechi and Albright, 2012)	7 ​y^a^ (Allemani et al., 2018; Bhakta et al., 2019; Stoeter et al., 2021; Stagno et al., 2014; Dewan et al., 2018; Grewal et al., 2020; Uche et al., 2021; Seah et al., 2019; Page et al., 2021; Central Nervous System Tumours, 2021; Aromataris and Munn, 2020; Core Team, 2020; Balduzzi et al., 2019; DerSimonian and Laird, 1986; Egger et al., 1997)	1 (100)	Intraventricular meningioma	Complete resection	Patient survived to follow-up (6 months)
Olufemi Adeleye (2009) (Olufemi Adeleye and Balogun, 2009)	16 ​y	1 (100)	Ivth ventricular medulloblastoma	Near-total resection	NA
Onyia (2020) (Onyia and Ojo, 2020)	8 ​y	0 (0)	Pylocytic astrocytoma	Subtotal resection	NA
Salami et al. (2019) (Salami et al., 2019)	8 ​y (8 ​m-17 ​y)	6 (67)	Meningioma	NA	Patient survived to follow-up (8 ​m–4 ​y)
Seligson (1974) (Seligson and Levy, 1974)	(2–15 ​y)	NA	Astrocytoma grade IV	NA	Patient died
Uche et al. (2021) (Uche et al., 2021)	10 ​y (7 ​m-16 ​y)	42 (Jibrin et al., 2018)	Craniopharyngioma (n ​= ​21) Medulloblastoma (n ​= ​17) Astrocytoma (n ​= ​12) Ependymoma (n ​= ​6) Meningioma (n ​= ​8) Oligodendroglioma (n ​= ​3) DNET (n ​= ​2) PNET (n ​= ​5) Pituitary adenoma (n ​= ​2) Hemangioblastoma (n ​= ​2)	Total microsurgical resection (n ​= ​26) Subtotal microsurgical resection (n ​= ​50)	51 (67%) survived 1 year 37 (49%) survived 5 years
Uche et al. (2013) (Uche et al., 2013)	10 ​y (10 ​m-15 ​y)	18 (El-Gaidi, 2011)	Low-grade astrocytoma (n ​= ​10) Medulloblastoma (n ​= ​10) High-grade astrocytoma (n ​= ​3) Craniopharyngioma (n ​= ​5) Pituitary adenoma (n ​= ​2) Supratentorial PNETs (n ​= ​2) Germ cell tumor (n ​= ​2) Oligodendroglioma (n ​= ​1) Meningioma (n ​= ​1) Ganglioglioma (n ​= ​1) Ependymoma (n ​= ​1) Epidermoid tumors (n ​= ​2)	Total resection (n ​= ​25) Subtotal resection (n ​= ​15)	17 (57%) survived 1 year 14 (47%) survived 5 years
Wanyoike (2004) (Wanyoike, 2004)	7 ​y (Pollack, 1994; Allemani et al., 2018; Bhakta et al., 2019; Stoeter et al., 2021; Stagno et al., 2014; Dewan et al., 2018; Grewal et al., 2020; Uche et al., 2021; Seah et al., 2019; Page et al., 2021; Central Nervous System Tumours, 2021; Aromataris and Munn, 2020; Core Team, 2020; Balduzzi et al., 2019)	24 (65)	Astrocytoma (n ​= ​11) Medulloblastoma (n ​= ​11) Ependymoma (n ​= ​3) Tuberculoma (n ​= ​3) Meningioma (n ​= ​1)	Extent unknown. Shunt (n ​= ​28) No shunt (n ​= ​5)	12 (40%) died (after mean 20 ​d, 0–66 ​d)
Wilson et al. (2012) (Wilson et al., 2012)	9 ​y	0 (0)	Medulloblastoma	Biopsy	NA

a Median.

b Patients who were included had >5 years of survival a priori.

### 3.3 Patient outcomes

Survival and mortality differed widely between studies, and the data were reported using variable formats (Table 3). Even within studies, there was large heterogeneity. The four largest studies (n ​= ​37–76 patients) included several tumor types each, preventing the reader from drawing tumor-specific conclusions related to surgical resection. Adeloye et al. investigated only craniopharyngioma in nineteen patients, for which they found that almost one-third of patients died within the first six months; however, the extent of resection was not specified, preventing comparison to other studies. Only four studies reported length of stay, but three of them were case studies. There were no trends identified in the reporting of the post-operative course and complications (Table 4). The only outcome that could be analyzed with a meta-analysis was 1-year survival, for the studies of Ndubuisi and both studies by Uche. However, since these three studies all have several different histological tumor types and different extents of resection, we refrain from presenting the results here since the clinical meaning is limited. Instead, the results are instead presented in Supplementary File 2 for the interested reader. Overall, considering the large amount of case reports and sub-setting of larger studies, the certainty of evidence from this compilation of data is low.

**Table 4:** Postoperative course.

Study	Post-operative recovery	Post-operative complications	Notes
Adeloye et al. (1988) (Adeloye et al., 1988)	Length of stay: median 30 ​d (Core Team, 2020) Of survivors: 4 showed general and visual improvement (headaches, personality changes, vision improvement); 8 did not improve	Postoperative diabetes insipidus (n ​= ​3) Neurological deficit (n ​= ​2) Meningitis (n ​= ​2) Recurrence (n ​= ​2)	
Adeolu et al. (2015) (Adeolu et al., 2015)	Satisfactory recovery (no neurological deficit 6 w after discharge)	None (at 6 w)	
Andrews et al. (2003) (Andrews et al., 2003)	Karnofsky performance status post-op was <70 for both patients (only one patient had <70 pre-op)	Not specified	
Anunobi et al. (2016) (Anunobi et al., 2016)	Died 6 ​h post-surgery	Fatal cardiac arrest after hyperthermia and tachycardia 6 ​h post-op	
Charles et al. (2019) (Charles et al., 2019)	Not specified	Recurrence after 9 ​m, died after re-operation	Parents did not adhere to adjuvant oncology follow-up
Elhassan et al. (2019) (Elhassan et al., 2019)	Not specified	Not specified	Only patients who survived at least 5 years were included in subset
Iddrissu et al. (2005) (Iddrissu et al., 2005)	Improvement of 6th nerve palsy slow (resolved after 12 ​m); normal mentation, mildly ataxic	None	
Kakusa et al. (2019) (Kakusa et al., 2019)	Not specified	Not specified	
Labuschagne (2020) (Labuschagne, 2020)	Not specified	Wound complications (n ​= ​2) Transient partial hemianopia (n ​= ​1) Transient worsening of preoperative hemiparesis (n ​= ​1) Recurrence (astrocytoma IV, n ​= ​2; embryonal tumor, n ​= ​1)	Follow-up median 13 ​m (Stagno et al., 2014; Dewan et al., 2018; Grewal et al., 2020; Uche et al., 2021; Seah et al., 2019; Page et al., 2021; Central Nervous System Tumours, 2021; Aromataris and Munn, 2020; Core Team, 2020; Balduzzi et al., 2019; DerSimonian and Laird, 1986; Egger et al., 1997; Begg and Mazumdar, 1994; Adeloye et al., 1988; Adeolu et al., 2015)
Malomo et al. (2018) (Malomo et al., 2018)	Good post-operative recovery; discharged 11 weeks after admission	Transient hyperthermia and tachycardia (hypothalamic dysfunction), resolved after 72 ​h with appropriate medication	No follow-up after discharge at 11 weeks
Mwang'ombe et al. (2002) (Mwang'ombe et al., 2002)	Post-operative cause was uneventful; discharged after 10 days	None	
Nadvi (1994) (Nadvi and van Dellen, 1994)	Initial recovery good; symptoms decreased (headache, neck stiffness, third nerve palsy, hallucinations)	Spinal metastases (3 w post-op); received radiotherapy	
Ndubuisi (2018) (Ndubuisi et al., 2018)	Outcome after 1 year: 2 (4.4%) dependent, 5 (11.1%) independent, 20 (44.4%) normal	Not specified	8 (14.8%) patients had "other" tumor, which could be non-primary brain tumor
Okechi (2012) (Okechi and Albright, 2012)	6 months postoperatively (last follow-up): asymptomatic and right hemiparesis completely resolved	Aseptic meningitis with subdural effusion, requiring drain; 3 ​m later new subdural effusion required second drain (relieved problem)	
Olufemi Adeleye (2009) (Olufemi Adeleye and Balogun, 2009)	Hearing impairment improved after 3 weeks, good after 5 months Remained blind bilaterally	None	
Onyia (2020) (Onyia and Ojo, 2020)	Initially he had transient neurologic symptoms (decreased consciousness, hemiparesis, right oculomotor palsy); slow but steady recovery Back to school with no other problems	None	
Salami et al. (2019) (Salami et al., 2019)	Good clinical recovery 1 impaired visual acuity had partial recovery of vision 1 seizure had a marked reduction in seizure frequency at last follow-up (8 ​m–4 ​y)	None	
Seligson (1974) (Seligson and Levy, 1974)	Not specified	In-hospital death	
Uche et al. (2021) (Uche et al., 2021)	38 (69%) did not finish school, and 46 (84%) did not finish school with passing grades (N ​= ​55, attending school initially) after surgery	Not specified	
Uche et al. (2013) (Uche et al., 2013)	Not specified	Not specified	10 lost to follow-up
Wanyoike (2004) (Wanyoike, 2004)	Not specified	Not specified	11 active follow-up, 7 lost to follow-up
Wilson et al. (2012) (Wilson et al., 2012)	Stable outcome	None	

## 4 Discussion

This is the first systematic review aimed at summarizing the outcomes after pediatric brain tumor surgery in Sub-Saharan Africa. Nigerian institutions have published most data on the topic. Among the included studies, commonly only subgroups of patients met the inclusion criteria, and there were a handful of studies that had inconsistent sample sizes among variables of interest. Furthermore, there were 9 (41%) case reports among the included studies. Among the remaining studies, only six (46%) included more than 10 patients. All these flaws considerably biased our potential findings due to the subjective patient recruitment. Additionally, surgical extent differed for certain PBT types, such as craniopharyngioma (Table 3), which may be resected totally, or sub-totally, the latter often followed by radiotherapy (Grewal et al., 2020). This may have affected heterogeneity in the reporting of outcomes. Overall, it was difficult to identify a general tendency among the surgical outcome of pediatric brain tumors in Sub-Saharan Africa (Table 3, Table 4).

Among the 46 countries that constitute the Sub-Saharan African region, only 8 countries (Nigeria, Ghana, Sudan, Uganda, Tanzania, Kenya, Rhodesia and South Africa) were represented among study locations (Fig. 2). This further highlights the scarcity of data on PBT and associated surgical outcomes in this region, which is consistent with previous studies that suggest a low access to high quality pediatric cancer registries (Bhakta et al., 2019) and research data (Stefan, 2015) in Sub-Saharan Africa. When reported, post-operative mortality in general was higher compared to those reported in HIC (Neervoort et al., 2010; Foster et al., 2021). In chart reviews, with more than 10 patients, 1-year post-operative mortality of PBT ranged from 33 to 43% (Table 3), while overall data on surgical mortality of PBT in HIC ranged from 0 to 20% (Neervoort et al., 2010). Previous studies have suggested that the higher overall PBT mortality rates in Sub-Saharan African countries and other LMIC, are the result of underdiagnosis (Stagno et al., 2014), and lack of access to neurosurgical care (Dewan et al., 2018; Park et al., 2021).

The three most common PBT types were medulloblastoma, craniopharyngioma and astrocytoma (Table 2). Although the scarcity of data prevents conclusions to be drawn regarding distribution of specific pediatric tumor types in this region, a cautious comparison to HIC (Pollack, 1994) indicate some similarities in distribution of medulloblastoma and astrocytoma. However, craniopharyngioma, a slow-growing, benign tumor, does not appear as common in HIC (Pollack, 1994), which may indicate some geographical differences or may be due to delayed presentation. Moreover, results are consistent with previous studies that have shown a large variation in PBT presentation between settings in LMIC (Asirvatham et al., 2011; El-Gaidi, 2011; Jibrin et al., 2018).

The main limitation of the evidence, is the restricted number of available research studies conducted on PBT in Sub-Saharan Africa and their surgical outcomes. However, PubMed was the only database used, and a more extensive search in multiple search engines, such as Scopus, EMBASE and African Index Medicus, would probably have yielded more literature. However, other databases were expected to primarily find additional smaller reports in the forms of conference abstracts, theses, non-medical texts, or other gray literature and non-peer-reviewed work. Even so, the search performed herein returned a high number of case reports, limited number of included patients, and sample size inconsistencies, all of which increase the risk of bias. On the other hand, not restricting the publication date resulted in a few old studies, including Seligson et al. (Seligson and Levy, 1974) from 1974 and Adeloye et al. (1988) from 1988, that have little clinical importance today but were included for completeness.

Considering that a meta-analysis was only mathematically possible but not clinically meaningful, it is clear that this first systematic review on the topic has identified the need for more research on PBT and associated treatment outcomes in Sub-Saharan Africa. Lacking research in a strained environment comes as no surprise; Sub-Saharan Africa has the lowest rate of neurosurgeons per capita in the world (Mukhopadhyay et al., 2019), and previous studies have emphasized the importance of investing in global neurosurgical care (Dewan et al., 2018; Meara et al., 2015; Park et al., 2016). One resource to improve research of small centers is the use of registries. Local and regional cancer registries would answer the specific questions posed in this study and many more, but they are still limited and rarely record surgical outcomes. With the ongoing establishment of electronic health records in the region (Odekunle et al., 2017), we encourage an expansion of the registries to include relevant surgical and oncologic data.

## 5 Conclusion

Data on outcomes after pediatric brain tumor surgery in Sub-Saharan Africa is insufficient and inconsistent, preventing any statistical conclusions to be drawn. There is an overrepresentation of Nigerian studies, while several countries are not represented. This systematic review highlights the need for more studies in the field.

## Contributions

VH: Design, data collection, analysis, writing the first draft, editing & reviewing, final approval.

PL: Design, data collection, analysis, writing the first draft, editing & reviewing, final approval.

FAB: Design, editing & reviewing, final approval.

JB: Design, editing & reviewing, final approval.

EU: Design, editing & reviewing, final approval.

MT: Design, analysis, editing & reviewing, final approval.

## Funding

This research did not receive any specific grant from funding agencies in the public, commercial, or not-for-profit sectors.

Philipp Lassarén acknowledges funding from the Swedish 10.13039/501100000942Brain Foundation (#FO2019-0006) for a laptop. The funders had no role in the design or conduct of this research.

## Declaration of competing interest

The authors declare that they have no known competing financial interests or personal relationships that could have appeared to influence the work reported in this paper.
