# Supplementary material for: Surgical outcomes of pediatric brain tumors in Sub-Saharan Africa: A systematic review
Source: Brain Spine. 2022 Jul 3;2:100912. doi: 10.1016/j.bas.2022.100912 (PMC9560713; doi:10.1016/j.bas.2022.100912)
Supplement: Multimedia component 1 [file mmc1.docx]

**Supplementary File 1. Excluded studies.**

Studies excluded because no full text was found: (1-11)

1. Adeolu AA, Adeniji AO, Komolafe EO, Amusa YB, Olateju SO, Oyebamiji EO, et al. Review of skull base surgery in a Nigerian teaching hospital. Niger Postgrad Med J. 2010;17(1):50-4.

2. Bankole OB, Ojo OA, Kanu OO, Arigbabu SO. Choroid plexus papilloma in a Nigerian child: a case report. Niger Postgrad Med J. 2010;17(3):233-6.

3. Binitie OP, Obikili AG. Pulsating orbital plexiform neurofibroma and optic nerve glioma. East Afr Med J. 1989;66(5):362-4.

4. Keita AD, Kane M, Guinto CO, Landoure G, Traore S, Karembe M, et al. [Using CT to diagnose brain tumors at the Point G Hospital in Mali]. Mali Med. 2007;22(2):14-8.

5. Moreira I, Pereira J, Oliveira J, Salvador SF, Vaz R. Endoscopic re-opening of third ventriculostomy: Case series and review of literature. Clin Neurol Neurosurg. 2016;145:58-63.

6. Mwang'ombe NJ, Ombachi RB. Brain tumours at the Kenyatta National Hospital, Nairobi. East Afr Med J. 2000;77(8):444-7.

7. Oyemolade TA, Shokunbi MT, Badejo OA, Adeolu AA. Brainstem Glioma: Clinical Profile and Challenges of Management in a Developing Country. West Afr J Med. 2019;36(2):172-5.

8. Shamboul K, Grundfast K. Hearing loss in neurofibromatosis type 1: report of two cases. East Afr Med J. 1999;76(2):117-9.

9. Sur RK, Nayler S, Ahmed SN, Donde B, Uijs RR, Cooper K, et al. Angiosarcomas--clinical profile, pathology and management. S Afr J Surg. 2000;38(1):13-6.

10. Togo P, Togo B, Doumbia AK, Traoré F, Touré A, Diakité AA, et al. Childhood brain tumors in Mali. Med Sante Trop. 2019;29(3):264-7.

11. Udoh MO. Central Nervous System Pathology in Children: A Single-Institution Experience in South-South Nigeria. West Afr J Med. 2022;39(2):134-9.

**Supplementary File 2. Meta-analysis.**

In order to be able to do a meta-analysis, each included study needs to report a statistical measure of spread, which means that it has to have at least three participants (so that a measure of spread can be estimated for the particular study). There are 8 studies with at least three participants. In order to meta-analyze on these studies, they need to be reporting on the same outcome/measure of interest and have similar patient groups. The only outcome which fulfils these criteria is 1-year mortality, with three papers reporting it. A forest plot is shown below. From the pooled data, 55 % of patients survive one year. There is large heterogeneity, as indicated by the high I2 value of 0.76.

Egger’s and Begg’s tests for publication bias were non-significant (p = 0.64 and p = 0.60, respectively). A similar result can be visualized in the funnel plot below.

However, the clinical usefulness of these results is limited, since the three studies contain different types of mixes of tumor types and various levels of surgical extent.
